# Unraveling the genetic basis of xylose consumption in engineered *Saccharomyces cerevisiae* strains

**DOI:** 10.1038/srep38676

**Published:** 2016-12-21

**Authors:** Leandro Vieira dos Santos, Marcelo Falsarella Carazzolle, Sheila Tiemi Nagamatsu, Nádia Maria Vieira Sampaio, Ludimila Dias Almeida, Renan Augusto Siqueira Pirolla, Guilherme Borelli, Thamy Lívia Ribeiro Corrêa, Juan Lucas Argueso, Gonçalo Amarante Guimarães Pereira

**Affiliations:** 1 Laboratório de Genômica e Expressão, Departamento de Genética e Evolução, UNICAMP, Campinas, São Paulo 13083-970, Brazil; 2 GranBio/BioCelere, Campinas, Brazil; 3 Department of Environmental and Radiological Health Sciences, Colorado State University, Fort Collins-CO, 80523-1618, USA

## Abstract

The development of biocatalysts capable of fermenting xylose, a five-carbon sugar abundant in lignocellulosic biomass, is a key step to achieve a viable production of second-generation ethanol. In this work, a robust industrial strain of *Saccharomyces cerevisiae* was modified by the addition of essential genes for pentose metabolism. Subsequently, taken through cycles of adaptive evolution with selection for optimal xylose utilization, strains could efficiently convert xylose to ethanol with a yield of about 0.46 g ethanol/g xylose. Though evolved independently, two strains carried shared mutations: amplification of the xylose isomerase gene and inactivation of *ISU1*, a gene encoding a scaffold protein involved in the assembly of iron-sulfur clusters. In addition, one of evolved strains carried a mutation in *SSK2*, a member of MAPKKK signaling pathway. In validation experiments, mutating *ISU1* or *SSK2* improved the ability to metabolize xylose of yeast cells without adaptive evolution, suggesting that these genes are key players in a regulatory network for xylose fermentation. Furthermore, addition of iron ion to the growth media improved xylose fermentation even by non-evolved cells. Our results provide promising new targets for metabolic engineering of C5-yeasts and point to iron as a potential new additive for improvement of second-generation ethanol production.

The global energy matrix continues to rely heavily on fossil fuels whose combustion releases large amounts of CO_2_ into the atmosphere, a potent greenhouse gas and a major contributor to climate change^1^,^2^,^3^,^4^. Bioethanol has been touted as a cleaner and renewable alternative to replace fossil fuels and reduce carbon emissions^5^. In Brazil, first-generation ethanol is produced from the fermentation of sucrose-rich sugarcane extract, using a process in which *Saccharomyces cerevisiae* yeast cells are continuously recycled through successive batches, in some cases for up to 250 days in a row^6^. This prolonged cell recycling regimen combined with the harsh environment found in the bioethanol distilleries require strains that display traits such as high tolerance to abiotic stress and robust growth to outcompete microbial contaminants. One of the most widely adopted strains, Pedra-2 (PE-2), was itself originally isolated as an aggressive wild contaminant^7^. It combines high fermentative capacity and environmental stress tolerance, with the ability to prevent other yeast invaders from getting established in the fermentation tanks, thus providing stability to the distilleries’ industrial operations. These desirable traits make PE-2 an ideal biological platform for the delivery of new technologies essential for second-generation (2 G) bioethanol production^8^,^9^.

A major challenge to the establishment of second-generation operations is the availability of biocatalysts able to metabolize the five-carbon (C5) sugar xylose derived from the hydrolysis of lignocellulosic biomass feedstocks^5^. *S. cerevisiae* is normally unable to metabolize xylose, but two pathways have been commonly used to engineer this activity into yeast cells^10^,^11^. One is the oxidative-reductive pathway, catalyzed by the enzymes xylose reductase (XR) and xylitol dehydrogenase (XDH). A more advantageous alternative is the xylose isomerase (*xylA*) pathway, which involves a single-step conversion of xylose to xylulose. The *xylA* enzyme does not require cofactors and therefore does not have the redox bottleneck associated with the XR-XDH pathway. In addition, since it has lower accumulation of by-products, the total amount of ethanol that can be produced is higher^10^,^12^,^13^,^14^.

The successful expression of a functional *xylA* in *S. cerevisiae* allowed the development of strains able to ferment xylose to ethanol^15^,^16^. However, the resulting strains showed low xylose consumption rates under anaerobic conditions, which are incompatible with industrial scale production. To enhance strain performance, some genetic modifications have been shown as beneficial. For example, overexpression of genes from the non-oxidative phase of pentose phosphate pathway (PPP), deletion of the aldose reductase gene *GRE3*^17^ and transporters for enhanced xylose uptake rates^18^. In addition, adaptive evolution in xylose medium is typically conducted in batch or chemostat cultivation cycles to achieve the best results^19^,^20^,^21^. Successful examples of strains developed through this approach have been reported^22^,^23^,^24^, however, the specific mutations responsible for the acquired ability to metabolize xylose have not been well characterized.

In this work, we used PE-2 as the platform for construction of yeast strains able to efficiently ferment xylose into ethanol. Applying a relatively short period of evolutionary engineering (from 3 to 12 batches), we were able to select strains capable of efficiently consuming xylose as a sole carbon source in anaerobic conditions. We report a detailed genomic and physiological characterization of the best-performing evolved strains, and demonstrate that the improved consumption of xylose is associated with a high copy-number of *xylA* and inactivation of the *ISU1* and *SSK2* genes. In addition, with a better comprehension of C5-metabolism, we point out iron as a new additive that can induce improvement on performance of xylose fermenting strains, thus, suggesting new directions to increase the economic viability of second-generation technologies.

## Results

### Metabolic engineering and adaptive evolution of xylose fermentation

In order to build a robust xylose-fermenting *S. cerevisiae* strain, we dissected a tetrad from the industrial diploid strain PE-2 and chose a *MATα* haploid spore (LVYA1) for further genetic manipulations. Cassettes containing genes of xylose pathway (Supplementary Fig. S1) were designed and their integration was targeted to regions in the vicinity of centromeres, based on the rationale that such loci might offer higher stability to the integrated constructs due to lower recombination rates relative to other regions of chromosomes^25^. The first cassette contained the eukaryotic codon-optimized *xylA* gene from *Orpinomyces* sp. was integrated near *CEN5*. Next, we deleted the *GRE3* gene encoding aldose reductase in order to decrease the production of xylitol^26^, and integrated two additional copies of xylulokinase (*XKS1*) near *CEN2* and *CEN8*. Furthermore, an additional copy of each of the genes encoding the four non-oxidative enzymes from the pentose phosphate pathway, transaldolase (*TAL1*) and ribose-5-phosphate ketol-isomerase (*RKI1*) were inserted near *CEN12*; and transketolase (*TKL1*) and ribulose-5-phosphate 3-epimerase (*RPE1*) were inserted near *CEN13*, resulting in the strain LVY27. All these genes were expressed under the control of different strong constitutive promoters of genes from the glycolytic pathway of *S. cerevisiae* (Table 1).

**Figure 1:**
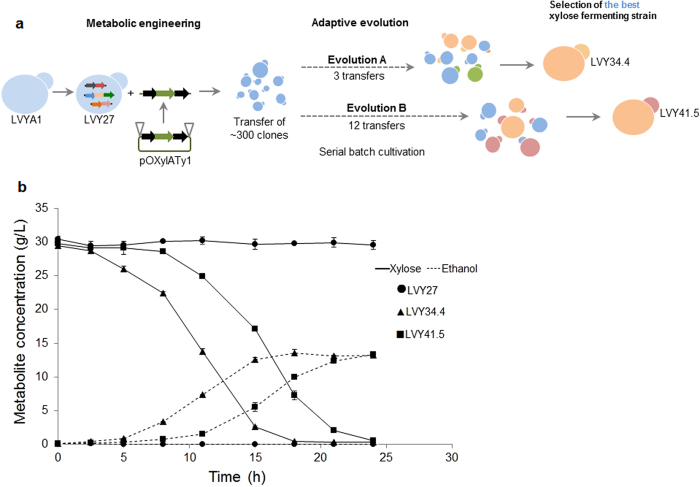
Development and comparative fermentation of evolved strains. (**a**) Flow diagram of genetic transformation and adaptive evolution steps. LVY27 strain was transformed with a DNA fragment containing the *xylA* gene (green arrow), which was flanked by two δ LTR regions (black arrows). Approximately 300 resulting clones were used in adaptive evolution experiments. After a first batch of cultivation, the cells were split into two parallel evolution experiments (**a** and **b**). The colors represent the mutations acquired by the strains during evolution. The fastest xylose fermenting strains from each evolution, LVY34.4 and LVY41.5, were selected for further investigation. (**b**) Comparative performance of strains in batch fermentation. Strains were cultivated in YP medium supplemented with 30 g/L of xylose with an initial cell density of 0.25 g DW/L of the parental LVY27 (●) and evolved cells LVY34.4 (▲) and LVY41.5 (■). The fermentations were performed in triplicate and error bars represent standard deviation from the average of values.

Despite integration of the entire xylose utilization pathway, the resulting strain LVY27 consumed xylose very slowly and growth was observed only under aerobic conditions. To achieve a higher activity of xylose isomerase, we applied an approach to facilitate amplification of the *xylA* gene. Plasmid pOXylATy1 (Supplementary Table 1) was created to incorporate a cassette containing *xylA* flanked by δ LTR sequences. The plasmid was cleaved and the purified fragment, containing the δ-*xylA*-δ fragment was used in a new integrative transformation (Fig. 1a).

Following the premise that cells containing the highest number of δ-*xylA*-δ fragment integrations would have a higher xylose consumption rate, ~300 colonies derived from the transformation of strain LVY27 were selected to undergo adaptive evolution in xylose medium. The different transformants were allowed to compete with each other in YNBX medium in semi-anaerobic conditions. Glucose was added to the medium to allow initial growth and the cells were split into two new sealed bottles and parallel adaptive evolution experiments (A and B) were started (Fig. 1a). After glucose depletion, the cells were maintained in xylose as a sole carbon source for 48 hours before the next transfer. We started to observe cellular growth within three serial batch cultivations in xylose for evolution A and after 12 transfers for the parallel evolution B (approximately 15 and 60 generations, respectively). From each evolution, 12 candidate colonies displaying the largest diameter in solid xylose medium were selected and their growth was screened to identify the best xylose fermenting strains. Based on their faster growth kinetics in xylose medium, strains LVY34.4 and LVY41.5 were selected as the most promising clones derived from evolution A and B, respectively (Supplementary Fig. S2A and S2B).

Initially, each selected clone was compared to the parental strain LVY27 in YP medium supplemented with 30 g/L of xylose. Under these conditions, the parental strain LVY27 was not able to consume xylose or grow, while LVY34.4 and LVY41.5 consumed all xylose in less than 24 hours, with maximum specific growth rate of 0.213 and 0.129 h^−1^, respectively. Both had final ethanol yields of approximately 0.46 g ethanol/g xylose, but LVY34.4 had higher xylose consumption rate (Fig. 1b, Table 2). Glycerol and xylitol were observed in negligible levels. Taken together, these results show that the evolutionary engineering process allowed the selection of two strains, able to efficiently consume xylose and produce ethanol. In order to determine the genetic mechanisms through which improved xylose utilization was achieved, we sequenced the whole genomes of parent LVY27 and both evolved strains. The sequencing reads were analyzed to detect both structural rearrangements detected through depth coverage and nucleotide polymorphisms in the evolved clones.

### Genomic characterization of evolved strains I: *xylA* amplifications

The analysis of copy number variation (CNV) was performed using the cn.MOPS program^27^, employing the read-depth profile from each strain generated by paired-end reads alignment into the reference genome (parental genome assembly – LVY27). The comparison of read-depth between parental and evolved strains revealed a substantial amplification of a 6,237-bp region containing the genes *xylA* and *LEU2* that was previously inserted into the parental genome with plasmid pOXylATy1. This region was amplified 36-fold and 26-fold during the adaptive evolution process for the LVY34.4 and LVY41.5 strains, respectively. The Supplementary Figure S3 plots read-depth coverage in this region for each evolved strain and demonstrates the large coverage variation in the *xylA* + *LEU2* genes for both clones, that was around 1.34 times higher for LVY34.4 than LVY41.5. To further investigate this amplified region we performed a comparative sequence analysis that identified a specific contig present only in the evolved cells that harbor a combination of *LEU2* terminator, the δ LTR element, and the *TDH1* promoter sequences. This specific combination was generated through the fusion of two cassettes from pOXylATy1, suggesting that a tandem amplification of this site occurred during the adaptive evolution process. Alternatively, the amplification of the cassette could have occurred concomitantly with the initial integration event.

**Figure 2:**
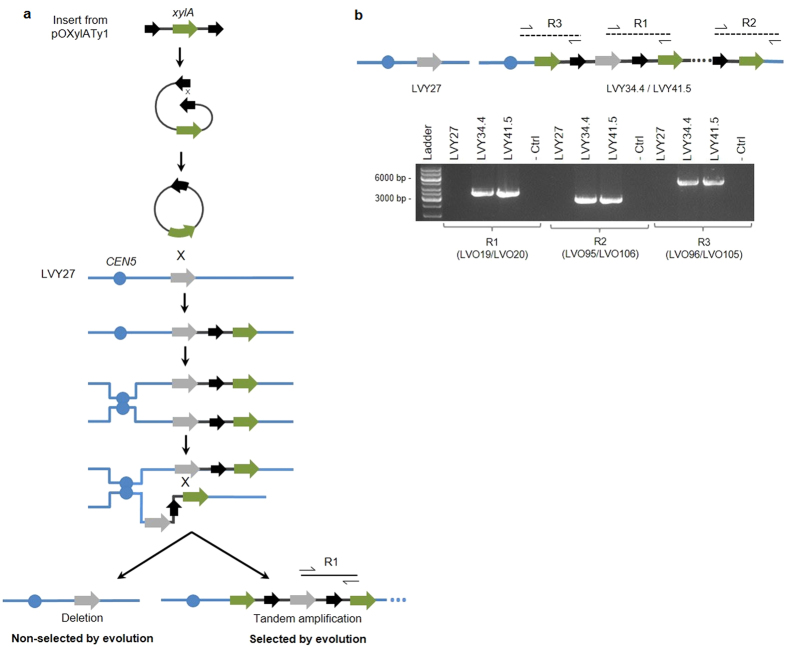
Potential mechanism that led to in tandem amplification of *xylA* in the evolved strains during evolution. (**a**) The blue strand represents a section of Chr05 in LVY27. The insert contains the *xylA* gene (green arrow) obtained from plasmid pOXylATy1 flanked by two δ LTR sequences (black arrows) in the same orientation. We hypothesize that shortly after the transformation event, the flanking LTR sequences from the DNA fragment recombined with each other creating a circular molecule. Next, a homologous recombination event occurred between the *xylA* sequence from the circle and the *xylA* sequence (represented by a gray arrow) that was previously inserted into the parental genome next *CEN5* region, resulting in the chromosomal integration of the circle. During duplication of the cell carrying this integration, unequal crossovers involving sister chromatids can result in tandem duplications of the insert containing *xylA*, thus leading to the amplification of this gene. Selection for growth in xylose allowed such *xylA* amplification events to increase in frequency in the cell population. (**b**) PCR reactions showing the tandem multiplication of *xylA* fragment during the evolution. The primers in the R2 and R3 reactions anneal with the Ty1 element (black arrow) and regions outside the integration locus. The R1 reactions amplified regions between two tandem cassettes. As expected, the reactions R1, R2 and R3 did not present positive amplification in LVY27. 1 Kb DNA ladder GeneRuler; (- Ctrl) control reaction without DNA.

In order to evaluate the stability of the transformants obtained, we retested the presence of each cassette containing the *xylA, XKS1* and other genes of the PPP into the genome of the evolved strains through PCR analysis. The presence of all cassettes was confirmed for all strains, except for the *xylA* cassette inserted near *CEN5*, whose presence was not detected in the strains LVY34.4 and 41.5 (Supplementary Fig. S4). By combining primers that anneal at the δ LTR region of plasmid pOXylATy1 with primers of regions flanking the integration site at *CEN5* (Fig. 2b, R2 and R3; Supplementary Table S2), we found that the cassette from pOXylATy1 recombined with the *xylA* already present in the genome of the strain LVY27 and integrated adjacent to that locus (Fig. 2a). In Fig. 2b, PCR reaction R1 used a combination of primers that annealed to the terminal region of each adjacent cassette. This product was excised from the agarose gel and sequenced. The sequencing analysis confirmed that both clones LVY34.4 and LVY41.5 contained tandem segmental amplifications of the *xylA* cassette in chromosome 5 (Chr05; Fig. 2b). Cells containing more copies of *xylA* likely had a growth advantage in xylose and succeeded under selective pressure (Fig. 2a).

**Figure 3:**
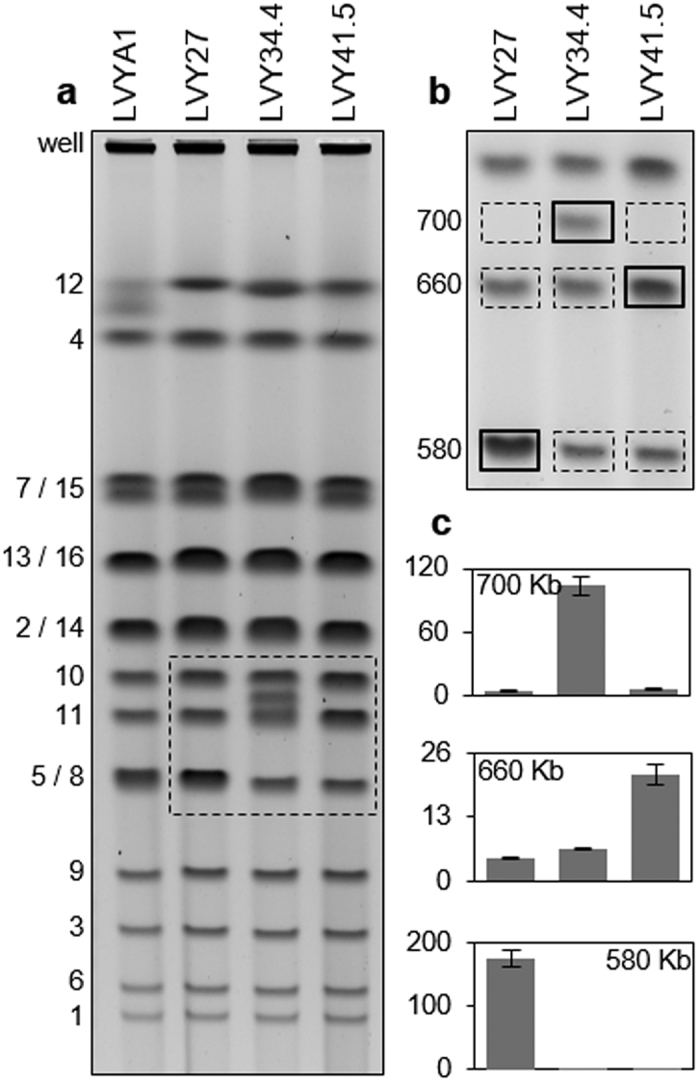
Karyotype analysis of evolved clones. (**a**) Pulse field gel electrophoresis (PFGE) under running conditions for optimal separation of all chromosomes. The sample loading well and bands corresponding to the wild PE-2 (LVYA1) and the parental strain LVY27 chromosomes are identified to the left. Yeast clones are indicated at the top of each lane. The dashed outline corresponds to the region of the PFGE karyotype analyzed at higher resolution in B. (**b**) Cropped PFGE under running conditions for maximum separation of molecules between 500 Kb and 800 Kb. Molecules larger than Chr10 ran as a single size-compressed band above the cropping point, and molecules smaller than Chr03 ran off the gel. Rectangles indicate the position of agarose slices excised from the gel, from which DNA was purified for qRT-PCR. Continuous outlines indicate gel slices with enriched Chr05 DNA in each lane, corresponding to the position of the parental size Chr05 in LVY27 (~580 Kb; 1 *xylA* repeat unit), and the rearranged Chr05 molecules containing segmental amplifications of the *xylA* insertion in LVY34.4 and LVY41.5 (700 Kb and 660 Kb, ~36 and ~26 repeat units, respectively). Dashed outlines correspond to gel slices without Chr05 DNA enrichment. (**c**) Plots of qRT-PCR results, showing the Chr05 DNA relative fold enrichment ratios (Y axes) in the gel slices. The bars corresponding to each clone appear directly under the respective gel lane from B. The size of the DNA fragments in the excised gel slice is indicated inside each plot.

To validate the presence of these chromosomal rearrangements we separated full-length chromosomes of the selected clones by Pulse Field Gel Electrophoresis (PFGE), using settings optimized to resolve the whole karyotype (Fig. 3a). We observed that LVY27 had the normal high intensity band at ~580 Kb that corresponds to the position where Chr05 and Chr08 co-migrate. In the LVY34.4 and LVY41.5 clones, we observed significant reductions in the intensities of the ~580 Kb band corresponding only to Chr08, indicating that Chr05 was present elsewhere in the PFGE. In LVY34.4, a new longer chromosome of ~700 Kb appeared, consistent with a ~120 Kb increase in size to Chr05 which would be expected for a tandem segmental amplification containing ~36 copies of the *xylA* cassette estimated from read depth coverage. Likewise, LVY41.5 had a higher intensity band at ~660 Kb, at the position where a single copy of Chr11 is normally found. This was consistent with a ~80 Kb increase in size to Chr05 due the presence of ~26 copies of the *xylA* cassette.

To refine our analyses of longer versions of Chr05 at those positions in LVY34.4 and LVY41.5, we changed the PFGE running conditions to maximize the separation of DNA molecules between 500 Kb and 800 Kb (Fig. 3b). Agarose slices were excised from these high resolution gels, and the DNA present in them was used as template for quantitative real time PCR (qRT-PCR; Fig. 3c). This analysis confirmed that Chr05 DNA was enriched relative to normalizing Chr09 and Chr16 sequences in the 580 Kb region of the parent strain LVY27 karyotype, but was not enriched in the two selected strains. Conversely, Chr05 specific DNA was enriched in the 700 Kb region in LVY34.4 and the 660 Kb region in LVY41.5. Taken together, the read depth, PCR, and karyotype analyses above confirmed that each of the two evolved clones carried tandem segmental amplifications of the *xylA* cassette insertion on Chr05.

### Genomic characterization of evolved strains II: Point mutations

The paired-end sequencing reads were assembled into large contigs (>1,000 bp) totaling 226 contigs (coverage, ~56×) for LVY27, 213 contigs (coverage, ~140×) for LVY34.4 and 232 contigs (coverage, ~128×) for LVY41.5. The whole genome sequencing results are summarized in Supplementary Table S3. The identification of single nucleotide polymorphisms (SNPs) was performed using GATK package^28^. We identified a total of 335, 362, 375 variations for parental, LVY34.4 and LVY41.5, respectively. Interestingly, the comparison of allelic frequencies of each SNP between LVY34.4 and LVY41.5 showed that the allelic frequency of SNPs in LVY34.4 was 1 as expected, while for LVY41.5 was always 0.5 (Supplementary Table S4). This result suggested that LVY41.5 potentially underwent a diploidization event during the adaptive evolution.

A customized PERL script was developed to identify polymorphisms that systematically appeared in the evolved strains and that, conversely, were absent in LVY27 (see methods). A total of nine SNPs and one indel were identified using this approach. These SNPs/INDELs were manually annotated using the reference genome of *S. cerevisiae* and filtered by mutations residing only inside coding sequences. This analysis resulted in a short list of potentially significant nucleotide mutations acquired during the adaptive evolution (Supplementary Table S4). Comparing the shared modifications, three non-synonymous SNPs were distributed in two genes: *ISU1* and *IKI3*. In addition, another polymorphism of interest, a non-sense mutation in *SSK2*, was identified in LVY41.5 strain.

### Functional analyses of nucleotide mutations

The strain LVY34.4 was initially chosen as the background to individually validate the two identified shared mutations at the *IKI3* and *ISU1* genes. Initially, the *URA3* gene was deleted in LVY34.4 so it could later be used as a selection marker, resulting in the strain LVY54 (Table 1; see methods). Both alleles of the *IKI3* gene were amplified either from the parental LVY27 strain (WT sequence) or the evolved LVY34.4 strain (*Ile398Val*), resulting in the strains LVY57, which contained the gene in its original form, and LVY58 that contained the mutated copy of the gene. The fermentative performance of LVY57 and LVY58 were evaluated and compared to the LVY54 control (LVY34.4 *ura3Δ*, Table 1). All strains displayed a lower rate of xylose consumption due to uracil auxotrophy. Under the tested conditions, we did not observe a significant difference in xylose consumption or ethanol production between LVY57 and LVY58 (Supplementary Fig. S5), suggesting that the SNP identified in *IKI3* was not related to the improved capacity for xylose consumption at the evolved strains.

**Figure 4:**
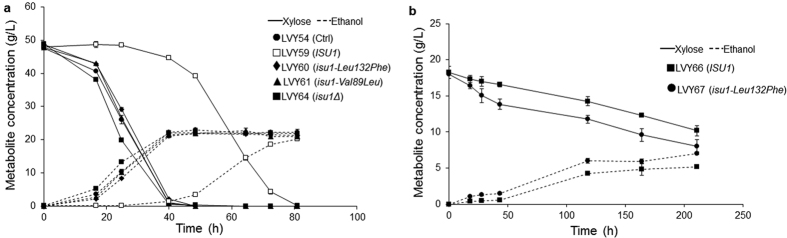
Fermentative performance of the transformants with *ISU1* SNPs in xylose medium. (**a**) Effect of *ISU1* mutations in the fermentative performance of strains. Strains were cultivated in YPX in batch fermentation with an initial cell density of 0.12 g DW/L. (**b**) Analysis of the effect of *ISU1* mutation in non-evolved cells. Strains were cultivated in YNBX. All the fermentations were performed in triplicate and error bars represent standard deviation from the average of values.

Next, we sought to investigate if the mutation found in the *ISU1* gene of evolved strains could be associated with the increased rates of xylose fermentation. Similarly to the approach above, *ISU1* was amplified from the genome of parental and evolved cells and used to originate LVY59, that harbored the gene in its original form; LVY60, which contained the allele from LVY34.4 (*Leu132Phe*); and LVY61, which contained allele found in LVY41.5 (*Val89Leu*) (Table 1). The xylose consumption rate in *ISU1* knockout strain LVY64 was also assessed. The LVY54 control strain consumed all the xylose in the medium in 40 hours, similar to those observed for LVY60 and LVY61. The two mutations had similar effects and no significant difference between the two strains was observed. The deletion of *ISU1* also enabled a similar consumption of xylose compared with the strains that have a mutation that substitutes an amino acid in Isu1p protein. In contrast, LVY59, which had the wild type *ISU1* allele, displayed very slow xylose consumption and sugar depletion was observed only after 80 hours (Fig. 4a). Taken together, these results confirmed that mutations that arose in *ISU1* during the adaptive evolution process contributed significantly to the enhanced fermentation of this sugar.

We also inserted the *isu1-Leu132Phe* allele in a strain that had not been evolved to isolate and test the effect of each mutation. To simulate the tandem amplification of the *xylA* gene, we introduced the multi-copy plasmid pOXylA2 (*URA3*) into LVY65 (LVY27 *ura3Δ*, Table 1). The fermentation was performed in YNBX medium without uracil. LVY67 consumes xylose slightly better than the cell with the wild type *ISU1* gene, LVY66 (Fig. 4b). However, the consumption rate for both strains in YNBX medium was much slower compared to YPX, even with *xylA* expressed from a high copy plasmid. We also isolated the effect of multiple copies of *xylA* by testing strain LVY68 with a single copy of *xylA* gene. As expected, the growth was very slow, suggesting that high expression of *xylA* gene is a necessary condition for efficient consumption of xylose (data not shown).

**Figure 5:**
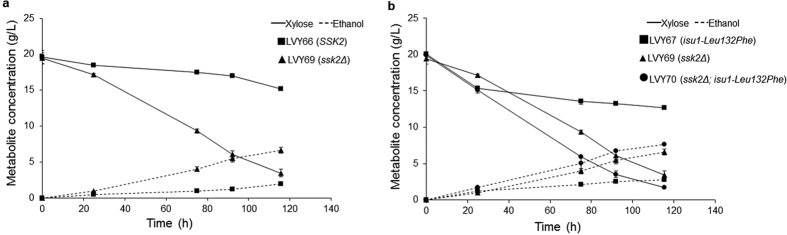
Comparative analysis of *SSK2* and *ISU1* mutations in non-evolved cells. Strains were cultivated in YNBX medium. (**a**) Effect of *ssk2* deletion. (**b**) The *ssk2* deletion confers a better xylose consumption rate than *isu1* mutation. The combinations of mutations give a better performance to double-mutant LVY70. All fermentation was performed in triplicate and error bars represent standard deviation from the average of values.

As previously mentioned, with the exception of *IKI3*, the SNPs found in the genomic analysis of LVY41.5 appeared at a frequency of 50% (Supplementary Table S4). This heterozygous pattern was not expected for *de novo* mutations that originated in a heterothallic haploid parent strain, thus suggested the occurrence of a diploidization event at some point in the LVY41.5 lineage during evolution. Using PCR^29^ analysis and flow cytometry^30^ (data not shown) we confirmed that LVY41.5 was in fact a diploid organism, but the mechanism that led to diploidization remained undetermined. LVY41.5 also contained a heterozygous nonsense mutation in the *SSK2* gene encoding a kinase member of the MAPKKK signaling pathway that regulates responses to osmotic stress conditions^31^. The *SSK2* gene was deleted in the parent strains as a single mutant, and in combination with the *isu1-Leu132Phe* mutation, resulting in LVY69 and LVY70, respectively. The strain containing only the *SSK2* deletion consumed xylose faster than control LVY66, confirming that this modification is also associated with xylose fermentation (Fig. 5a). Furthermore, in comparison to *isu1* mutation, LVY69 consumed xylose more efficiently and the *ssk2Δ isu1-Leu132Phe* double mutant LVY70 had an even better result, demonstrating a synergistic interaction between to these two pathways toward enhancement of xylose metabolism (Fig. 5b).

### Iron supplementation contributes to increased xylose fermentation

**Figure 6:**
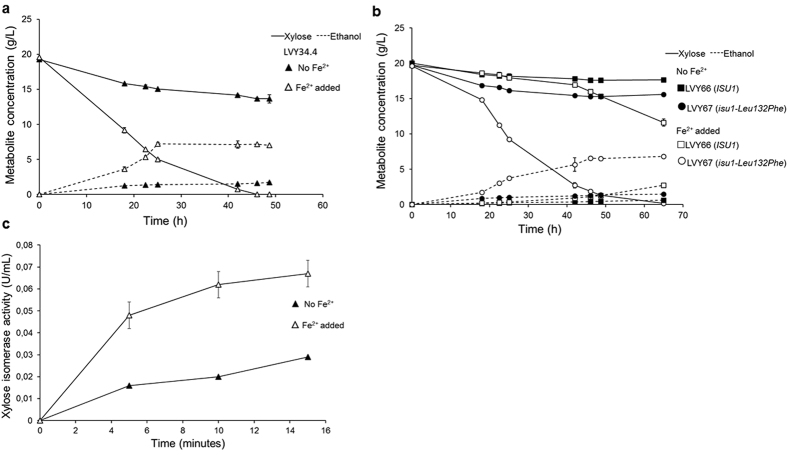
Iron supplementation effect on xylose fermentation. Effect of iron supplementation in YNBX on xylose consumption and ethanol production for LVY34.4 (**a**) and non-evolved strains LVY66 (*ISU1*) and LVY67 (*isu1-Leu132Phe*) (**b**). All fermentation was performed in triplicate and error bars represent standard deviation from the average of values. (**c**) Xylose isomerase activity of LVY34.4. The effect of Fe^2+^ ion was measured at 30 °C. The average of three replicates and standard deviations are presented.

Disruption of mitochondrial iron-sulfur clusters assembly results to increased accumulation of intramitochondrial iron concentrations^32^,^33^. Based on the hypothesis that increased levels of iron leads to increased xylose consumption, new fermentation assays were performed in YNBX medium supplemented with iron for strains described above. The evolved LVY34.4 were able to consume xylose and produce ethanol remarkably faster in the presence of increased concentration of iron ions, supporting the connection between the ability to consume xylose and the changes in iron metabolism observed in the evolved strains (Fig. 6a). The possible influence of other cofactors for the xylose isomerase activity such as Mg^2+^ and Mn^2+^ were also tested and did not result in improved growth in xylose (data not shown). The impact of iron ion was also tested on non-evolved cells. In this case, iron supplementation was also able to improve the consumption rate, although the strains harboring the *isu1-Leu132Phe* mutation had a better performance probably due to the higher capacity of accumulate iron intracellularly (Fig. 6b). The same effect was observed for the strains with *SSK2* deletion (Supplementary Fig. S6). Concomitantly, the addition of iron to reaction containing extracts of LVY34.4 strain significantly increased the xylose isomerase activity (Fig. 6c).

### Homozygous and heterozygous effect of *ISU1* mutation

LVY41.5 was subjected to a new round of adaptive evolution. After 11 transfers, the derivative strain LVY41.5EVx showed an increase rate of 2.7 times in xylose consumption compared to its immediate parent (Supplementary Fig. S7). As the LVY41.5 is a heterozygous *ISU1/isu1-Val89Leu*, the *ISU1* locus was sequenced in both strains. We observed two peaks in the LVY41.5 chromatogram corresponding to each allele of the parental spore. In LVY41.5EVx, only one peak was observed, indicating that this strain had become homozygous *isu1-Val89Leu/isu1-Val89Leu*. Therefore, the increase in the xylose consumption rate was due to the *ISU1* locus becoming in homozygous after second round of evolution. The *SSK2* mutation remained heterozygous.

**Figure 7:**
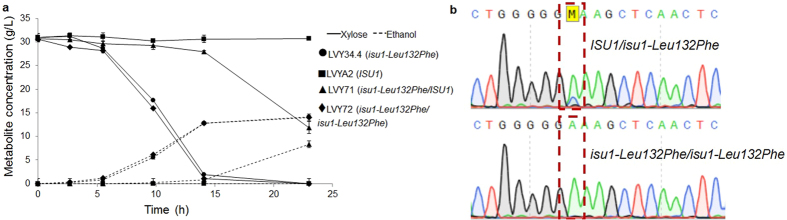
Homozygous and heterozygous effect of *ISU1* mutation in evolved and non-evolved diploid strains. Strains were cultivated in YP medium supplemented with xylose in a comparative batch fermentation (**a**) The haploids LVYA2 (■) and LVY34.4 (●) were used as controls. The non-evolved diploid LVY71 (▲) was compared to the evolved diploid LVY72 (◆), homozygous for *isu1* SNP. The fermentations were performed in triplicate and error bars represent standard deviation from the average of values. (**b**) Analysis of the top chromatogram from LVY71 indicated the presence of two peaks originating from both copies of the haploid spores LVYA2 and LVY34.4; while the evolved LVY72 bottom chromatogram from *ISU1* gene presents only one peak indicating the duplicate mutation by exchange of nucleotide at position 396 in both homologous chromosomes occurred during the second round of evolution.

To further investigate this effect, we crossed our best xylose fermenting strain LVY34.4 (*MATα*) with a *MATa* wild type haploid spore from PE-2 (Table 1). The resulting diploid was named LVY71 and its xylose fermenting capacity was compared to the parental spores in YPX. As expected, no growth was observed for the wild type spore, while LVY34.4 consumed all the sugar in 13 hours (Fig. 7a). The diploid was able to consume xylose, however, with very slow growth. LVY71 was heterozygous *ISU1/isu1-Leu132Phe*. With the premise that this is a recessive loss-of-function mutation, LVY71 was subjected to another round of adaptive evolution. The adaptation took twelve transfers and the evolved diploid LVY72 was chosen as the best xylose fermenting strain. LVY72 consumed xylose faster than its non-evolved version, at a rate comparable to the haploid LVY34.4 (Fig. 7a). The *ISU1* locus was sequenced in both strains and a similar sequence result was found. In the evolved strain LVY72, only one peak was observed, indicating that the strain had become *isu1-Leu132Phe/isu1-Leu132Phe*, confirming that homozygous *isu1* mutation ensures a more efficient consumption of xylose (Fig. 7b). We have seen that this was a recurrent phenomenon. We have repeated this procedure by crossing LVY34.4 with 4 haploid cells derived from the industrial strains CAT-1, SA-1, BG-1^7^ and CAETE-1 (isolated form a mill in the northeast of Brazil). In all cases, when the evolution led to an improved xylose consumption, we have identified that the *ISU1* wild type allele was lost, and the strains were homozygous for the *isu1-Leu132Phe* allele.

## Discussion

In this work, we have used the industrial strain PE-2 as the genetic background for the construction of a robust xylose-fermenting microorganism. Promoters of three genes that are highly expressed during the industrial 1 G fermentation^34^ were combined with coding sequences of 6 genes involved in xylose metabolism. Before construction, the codon usage of *Orpinomyces* sp xylose isomerase^35^ - the main specific enzyme of the xylose metabolism - was optimized for a more efficient expression in *S. cerevisiae*.

All the metabolized xylose is channeled through PPP to the glycolytic pathway for production of ethanol. However, even with the complete xylose consumption pathway integrated and overexpressed, the developed strain LVY27 consumes xylose poorly. Since an efficient expression of xylose isomerase is one of the main metabolic bottlenecks to use this pathway, the cassette containing the *xylA* was reconstructed using the δ LTR element, which could increase the number of integrations as described elsewhere^36^. Adaptive evolution of the engineered strain in xylose allowed a rapid improvement of the fermentative performance. In a very short time, we were able to select cells that efficiently fermented xylose. We found that in the selected strains that presented the highest fitness, LVY34.4 and LVY41.5, the integration of the second *xylA* expression cassette did not occur by homologous recombination with the retrotransposon Ty1 or solo δ LTRs elements. Instead, the single copy of the *xylA* sequence, already present in the host strain, was the actual site of integration (Fig. 2a and b). The best evolved strain LVY34.4 presents a yield of 0.46 g ethanol/g xylose (0.51 g/g is the *theoretical maximum*), similar or superior to most recently developed xylose-fermenting yeasts described in the literature^22^,^23^,^24^.

The genome analysis of the evolved cells revealed a large increase in the copy number of the gene encoding xylose isomerase. Recently, it was reported the generation of tandem repeats of *xylA* gene through a self-replicating extrachromosomal circular DNA (eccDNA) during adaptive evolution^37^. Considering that the *xylA* has a low activity in *S. cerevisiae*, the amplification during evolution seems to be an essential condition to achieve high enzymatic efficiency. The same evidence was observed before, through multiple integration of *xylA* in the evolved strain^38^. A cell that harbors higher copy number of *xylA* during adaptive evolution in xylose media would convert this sugar faster, being positively selected (Fig. 2a). The difference in the copy number of *xylA* might partly explain the difference of productivity between LVY34.4 and LVY41.5 (Table 2).

In addition to the amplification of *xylA*, we identified some SNPs in evolved cells. As the replacement of the SNP discovered in *IKI3* resulted in no difference in xylose fermentation, this modification was determined not to be related to C5 metabolism (Supplementary Fig. S5). In the case of *ISU1*, the mutation appeared in different regions of the gene in the evolved cells isolated in separate experiments, suggesting a strong selective pressure in this gene for xylose adaptation. Isu1p is a conserved protein of mitochondrial matrix that performs a scaffolding function for the *de novo* synthesis of Fe/S clusters^39^. Iron/sulfur clusters (ISC) are essential cofactors for proteins involved in electron transport, enzymatic catalysis and regulation of several cellular processes^33^. The ISC system consists of a machinery of 17 proteins for Fe/S cluster assembly^40^. The ferrous iron is imported from cytosol to mitochondria through the inner membrane carriers Mrs3p-Mrs4p using proton motive force with the assistance of the glutaredoxins Grx3p-Grx4p as iron donors that facilitate the import^41^. The cysteine desulfurase complex Nfs1p-Isd11p donates the sulfur and cooperates with the frataxin Yfh1p that facilitates the delivery of ferrous iron to the scaffold protein Isu1p for [2Fe-2S] biogenesis^42^,^43^. In the second step, the co-chaperone Jac1p recruits the holo-form of Isu1p and conducts to the ATP-bound form of the mitochondrial Hsp70 chaperone Ssq1p. The binding of Ssq1p occurs in the LPPVK sequence of Isu1p, which triggers its ATPase activity inducing a conformational change that stabilizes the interaction and induces the dissociation^40^,^41^,^44^. In the evolved strain LVY34.4, the *isu1* mutation occurred at the LPPVK sequence, by changing a leucine for a phenylalanine at position 132. The mutation at this site and at position 89 in strain LVY41.5 probably eliminated the functionality of this protein, since both strains with these mutations showed similar phenotypes in comparison to LVY64 carrying a full *ISU1* gene deletion (Fig. 4a). The deletion of *ISU1* or the paralog *ISU2* have been reported to increase mitochondrial iron levels as well as reduce activity of [4Fe-4S] aconitase enzyme. Simultaneous deletion of both *ISU1* and *ISU2* is lethal to yeast cells^32^. In addition, yeast cells with depletion of other proteins of the Fe/S cluster scaffold machinery, such as *YFH1, NFS1*, or the chaperones involved also show increased concentrations of iron and mitochondrial oxidative damage^33^.

In LVY41.5, we identified a new mutation in the *SSK2* gene that contributes for higher xylose consumption than *isu1* mutations. This phenotype was confirmed through *SSK2* deletion. Remarkably, the combination of mutations in *SSK2* and *ISU1* conferred the best result obtained. The LVY41.5 became diploid during the course of evolution, even PE-2 being a heterothallic strain. The mutation found in LVY41.5 generated a stop codon at *SSK2* and was identified only in heterozygous state. The Sks2p protein interacts with Hog1p by direct phosphorylation to activate HOG pathway, which is a regulatory mechanism involved in the maintenance of cation homeostasis by signalizing for the plasma membrane alkali metal cation transporters^45^. Also, there is evidence that HOG pathway leads to the activation of regulatory response under high iron concentration^46^.

Therefore, mutations in genes of the ISC system, especially *ISU1*, significantly contribute to increase the iron ion inside yeast cells. These results could be correlated with high performance in xylose consumption, considering that xylose isomerase is a metalloenzyme that uses divalent cations as cofactor such as Mg^2+^, Mn^2+^, Co^2+^, and iron cations (Fe^2+^), although the latter is not the preferred cation^47^,^48^,^49^. According to the data displaced above, the SNPs probably led to an increase of cellular iron concentration, boosting the activity of xylose isomerase given that it acts as xylose isomerase cofactor (Fig. 6c). However, other possible consequences of changes in iron metabolism, besides the increased *xylA* activity, cannot be eliminated since Fe/S clusters are essential cofactors for several cellular processes^33^. Supporting the initial model, we observed a surprising effect in increasing of xylose consumption by LVY34.4 and other strains when iron was supplemented to YNBX medium (Fig. 6a). Iron supplementation also improves xylose consumption of not evolved strains (Fig. 6b and Supplementary Fig. S6). This result suggests the use of this cofactor as a potential supplement to increase the fermentation efficiency and boost the economic viability of 2G-ethanol production.

In summary, our work proved that specific mutations in the *SSK2* and *ISU1* genes increase xylose fermentation and, consequently, ethanol production by engineered *S. cerevisiae* strains. Surprisingly, both genes converge to pathways that are not related to carbohydrate catabolism. These data shows that the engineering of *S. cerevisae* strains to consume xylose and enable the production of 2G-ethanol are related to a not obvious complex metabolic network, which provides subsidy for many surprising discoveries in the field of second-generation ethanol.

## Methods

### Yeast strains and growth conditions

The *S. cerevisiae* strains used in this work are described in Table 1. Yeast sporulation and tetrad dissection were carried out using standard procedures^50^. YP medium (10 g/L yeast extract, 20 g/L peptone) supplemented with 20 g/L D-glucose (YPD) was used for inoculum preparation and yeast cells were cultivated at 30 °C, 200 rpm. Transformants were selected in YNB medium (6.7 g/L yeast nitrogen base without amino acids, Difco), supplemented with 1 g/L drop-out without uracil or leucine (Sigma), 20 g/L glucose or xylose and 20 g/L agar. For counter-selection, the transformants harboring the pSH65^51^ plasmid were cultivated in YNB supplemented with 440 mg/L uracil and 1 g/L 5-FOA. For antibiotic resistance, 200 mg/L of geneticin and 200 mg/L of hygromycin was added to YPD for strains expressing the *Kan*MX4^52^ or *hph*^53^ markers, respectively. Likewise, 300 μg/ml of zeocin was added for strains transformed with pSH65^51^.

### Plasmids

Expression cassettes were assembled in a single reaction^54^, using the pRS304^55^ or pRS426^56^ vector linearized with *Bam*HI. The fragments were amplified using Phusion DNA polymerase (NEB) with the primers described in Table S2. *xylA* gene sequence from *Orpinomyces* sp. was codon optimized for expression in *S. cerevisiae* (Eurofins) (supplementary material). The other genes, promoters and terminators were amplified from *S. cerevisiae* strain LVA1. All cassettes use *URA3* as marker flanked by two *loxP* sites. The main components of the plasmids are summarized in Table S1.

### Strains construction

The molecular techniques were performed using standard procedures^57^. Amplicons were purified from agarose gel using the kit Wizard® SV Gel and PCR Clean-Up System (Promega). The expression cassettes were amplified by PCR and used to transform yeast cells using the LiAc/SS-DNA/PEG protocol^58^. *URA3* gene was deleted from the LVA1 genome using *Kan-loxP* marker^52^ (G418 resistance), which was subsequently excised with pSH65 (*Cre-loxP* system)^51^. *GRE3* was deleted by replacement with *URA3* flanked by *loxP* sites and subsequent excised. The plasmid pOXylATy1 was cleaved with *Bam*HI and the resulting fragment was employed to transform LVY27, using *LEU2* as auxotrophic selection marker.

For functional analysis of mutations, the promoter and terminator sequences of *IKI3* and *ISU1* were fused to both ends of *URA3* and used to replace and delete the native *IKI3* and *ISU1* genes from LVY54′s genome (LVY34.4, *ura3Δ*), resulting in the Ura + strains LVY55 and LVY56, respectively (Table 1). The same procedure was used in the non-evolved cell LVY65 (LVY27, *ura3Δ*), to individually test the effect of each mutation. In this case, the plasmid pOXylA2 was inserted to simulate the high copy-number integration of *xylA*. For functional SNP validation, the *IKI3* and *ISU1* genes were amplified from the genomic DNA of LVY27, LVY34.4 and LVY41.5 and the purified PCR product was used to transform the evolved LVY55 and LVY56 and the non-evolved LVY65 strains to restore the genes at the same initial locus. The presence of each allele was confirmed by Sanger sequencing. *SSK2* was inactivated by insertion of hygromycin gene amplified from pAG32^53^.

### Adaptive evolution and selection of xylose-fermenting strains

Approximately 300 colonies derived from LVY27, transformed with the fragment obtained from the plasmid pOXylATy1, were inoculated in semi-anaerobic conditions into a 100 mL sealed bottle with YNB medium with 40 g/L of xylose, and incubated at 30 °C/200 rpm. Since no growth was observed using xylose as sole carbon source, glucose was added to a concentration of 0.5%. After the first round of evolution, the yeast cells were separated into two parallel experiments (Fig. 1a). If the culture showed growth, glucose was no longer added and a 5 mL aliquot was transferred to a new flask after the culture reached an OD_600nm_ of approximately 3. Three and twelve sequential batch cultivations were carried out for LVY34.4 and LVY41.5, respectively. The same procedure was employed for the diploid LVY71, but without addition of glucose. Culture samples were streaked on plates with solid xylose medium. Colonies showing the largest diameter were evaluated. The fastest growing strains LVY34.4 and LVY41.5 were selected for further characterization.

### Sequencing and genome assembly

Whole-genome sequencing of LVY27, LVY34.4 and LVY41.5 was performed using Illumina MiSeq at University of North Carolina (UNC, USA) sequencing facility. For each sample, millions of 2 × 300-bp paired-end reads were generated with an average size of 500 bp. The software PEAR^59^ was used to merge the overlapping paired-end reads in a single sequence. For each strain, overlapped reads were assembled into longer contigs using VELVET assembler^60^ with k-mer parameter optimized to maximize the N50 metric. The complete dataset of DNA-seq reads have been deposited in SRA under accession number SRP078504.

### Copy Number Variation (CNV) and Single Nucleotide Polymorphism (SNPs) analysis

The reads from each strain were mapped into the reference genome (LVY27 assembly) using Bowtie2 aligner^61^. The resulting BAM files were ordered using Picard package^62^ and used as input for cn.MOPS program^27^ to perform CNV analysis considering a window size of at least 1 Kb and a read coverage variation >2×. The command DepthOfCoverage available in the GATK program^28^ was used to extract from BAM files the read-depth coverage for each base pair. These values were normalized by its median to represent the CNV.

The SNP/INDEL detection for each strain was performed by GATK program using default parameters for base quality recalibrator, haplotype caller and variant filtration steps that processed the BAM files and generated three VCF (variant call format) files. Homemade PERL scripts were developed to process and compare the VCF files identifying SNPs/INDELs that appeared in the evolved strains and were absent in LVY27. Resulting SNPs/INDELs were submitted for manual annotation using 150 bp flanking region of each SNP/INDEL, extracted from evolved genome assembly, and BLASTing against *S. cerevisiae* genome using SGD database^63^.

### Molecular karyotyping analysis

PFGE procedure for full karyotype resolution was conducted as previously described^64^. DNA-embedded agarose plugs were loaded into 1% contour-clamped homogeneous electric field gels (CHEF) and gels were run in a BioRad CHEF-DR II system. Running conditions were as follows: 54 hours run time, 5 Volts/cm, initial and final switch times of 47 and 170 seconds, respectively. PFGE optimized for maximum resolution of molecules between 500 Kb and 800 Kb was conducted similarly, except for changes in total run time (84 hours), and initial and final switch times of 60 and 75 seconds, respectively. Slices were excised at sizes 580 Kb, 660 Kb and 700 Kb, purified using Thermo Scientific GeneJET Gel extraction Kit and the eluted DNA was used as template for qRT-PCR. The reactions were performed using PerfeCTa SYBR^®^ Green Fast Mix (Quanta BioSciences) on a BioRad CFX Connect Real Time System obeying this conditions: 30 s at 95 °C; 3 s at 95 °C; 15 s at 55 °C; 10 s at 72 °C; optical detection, repeat 34 cycles of steps 2 through 5. The amplification kinetics of three DNA sequence targets were determined: a segment of Chr05 outside of the *xylA* insertion region, and segments used for normalization from Chr09 (450 Kb) and Chr16 (920 Kb). The ΔCt between the Chr05 and Chr09 target was determined for each sample as a measure of Chr05 DNA template enrichment (A); The ΔCt between Chr16 and Chr09 was determined as a measure of enrichment of unspecific DNA templates (B); The A/B enrichment ratio was calculated and used as a measure of the abundance of Chr05 DNA templates in the slices relative to the abundance of unspecific DNA templates. Determinations were made in triplicate. The A/B ratios were averaged and the standard error calculated (Fig. 3c).

### Ploidy determination by flow cytometry

Diploidization of LVY41.5 was confirmed by flow cytometry, according described in elsewhere^30^. PE-2 and LVY34.4 were used as diploid and haploid controls, respectively. Exponentially growing cells were fixed in ethanol 70%. Cells were treated with RNase 2 mg.ml^−1^ and DNA was stained with propidium iodide 0.5 mg/ml in PBS buffer and incubated overnight. Fluorescence was analyzed with Flow cytometer FACS CANTO II.

### Enzyme assay

LVY34.4 cells grown in YPD medium and collected from mid-log phase were washed with sterile ice-cold water, centrifuged and resuspended in Y-PER reagent (Thermo Fisher Scientific). Xylose isomerase activity in LVY34.4 cell extracts was determined as described elsewhere^65^ adapted for microplate assay, using 100 mM Tris-HCl (pH 7.5) buffer, 0.001 mM FeSO_4_.7H2O, 0.15 mM NADH, 2 U sorbitol dehydrogenase (Sigma-Aldrich), and the cell lysate. The reaction started with the addition of 500 mM xylose. The activity (U/L) was determined by monitoring the oxidation of NADH at 340 nm during 15 minutes at 30 °C. One unit equals 1 μmol of substrate converted per minute under the conditions of the assay.

### Fermentation conditions

Semi-anaerobic batch fermentations were performed in YP medium supplemented with 30 g/L or 50 g/L of xylose as sole carbon source and the culture was started with a dry cell weight of about 0.25 g DCW/L. For functional analysis of SNPs, fermentations were carried out in YNB medium (without uracil) supplemented with 20 g/L of xylose, with initial cell weight of 0.12 g DCW/L. For iron supplementation tests, FeSO_4_ was added at a concentration of 100 μM at YNBX medium. For magnesium and manganese supplementation tests, MgSO_4_ and MnSO_4_ were added in concentrations of 500 μM and 100 μM, respectively. The fermentations were performed in 100 mL sealed bottles with working volume of 80 mL and incubated at 30 °C/200 rpm. Samples were taken to measure OD and subsequent analysis by HPLC. For cell dry weight determination, cells were collected by centrifugation, washed twice and dried in 65 °C. All experiments were performed in triplicate.

### Analytical procedures

Quantification of glucose, xylose, xylitol, glycerol, acetic acid and ethanol, was carried out by high-performance liquid chromatography (HPLC) using chromatograph Alliance (Waters) with refractive index detector (Waters 2414) and phothodiode array detector (Waters 2998) at 280 nm. The samples were analyzed by HPLC-RI-PDA using ion exclusion HPX-87H column (300 mm × 7,8 mm, BioRad^®^), heated in an oven at 35 °C, a 5 mM H_2_SO_4_ solution as the mobile phase at a flow 0.6 mL/min. A standard curve with known concentrations of compounds of interest was also analyzed using the same procedure.

## Additional Information

**How to cite this article**: Santos, L. V. *et al*. Unraveling the genetic basis of xylose consumption in engineered *Saccharomyces cerevisiae* strains. *Sci. Rep.* **6**, 38676; doi: 10.1038/srep38676 (2016).

**Publisher's note:** Springer Nature remains neutral with regard to jurisdictional claims in published maps and institutional affiliations.

## Supplementary Material

Supplementary Material

## Figures and Tables

**Table 1:** Yeast strains used in the study.

Strain	Parent strain	Relevant genotype/features	Reference/source
PE-2		Industrial ethanol production strain PE-2; *MAT*a/α	CBMAI 0959
CAT-1		Industrial ethanol production strain CAT-1; *MAT*a/α	CBMAI 0957
BG-1		Industrial ethanol production strain BG-1; *MAT*a/α	7
SA-1		Industrial ethanol production strain SA-1; *MAT*a/α	CBMAI 1547
Caeté-1		Industrial ethanol production strain Caeté-1; *MAT*a/α	Usina Caeté S.A.
LVYA1	PE-2	*MAT*α	This study
LVYA2	PE-2;	*MAT*a	This study
LVY27	LVYA1	*MAT*α; *CEN5::pTDH1-xylA-tTDH1*; *gre3Δ*; *CEN2::pADH1-XKS1-tADH1*; *CEN8::pADH1-XKS1-tADH1*; *CEN12::pTDH1-TAL1-tTDH1-pPGK1-RKI1-tPGK1*; *CEN13::pTDH1-TKL1-tTDH1-pPGK1-RPE1-tPGK1*	This study
LVY34.4	LVY27	*MAT*α; pOXylATy1 + adaptive evolution and selection	This study
LVY41.5	LVY27	*MAT*a/α; pOXylATy1 + adaptive evolution and selection	This study
LVY41.5EVx	LVY41.5	*MAT*a/α; adaptive evolution and selection	This study
LVY54	LVY34.4	*MAT*α; *ura3Δ*	This study
LVY55	LVY54	*MAT*α; *iki3Δ::URA3*	This study
LVY56	LVY54	*MAT*α; *isu1Δ::URA3*	This study
LVY57	LVY55	*MAT*α; *ura3Δ::IKI3*	This study
LVY58	LVY55	*MAT*α; *ura3Δ::iki3-Ile398Val*	This study
LVY59	LVY56	*MAT*α; *ura3Δ::ISU1*	This study
LVY60	LVY56	*MAT*α; *ura3Δ::isu1-Leu132Phe*	This study
LVY61	LVY56	*MAT*α; *ura3Δ::isu1-Val89Leu*	This study
LVY64	LVY54	*MAT*α; *isu1Δ*	This study
LVY65	LVY27	*MAT*α; *ura3Δ*	This study
LVY66	LVY65	*MAT*α; pOXylA2	This study
LVY67	LVY66	*MAT*α; pOXylA2; *isu1-Leu132Phe*	This study
LVY68	LVY27	*MAT*α; *isu1-Leu132Phe*	This study
LVY69	LVY66	*MAT*α; pOXylA2; *ssk2Δ*	This study
LVY70	LVY67	*MAT*α; pOXylA2; *ssk2Δ*; *isu1-Leu132Phe*	This study
LVY71	LVY34.4 x LVYA2	*MAT*a/α; *isu1-Leu132Phe/ISU1*	This study
LVY72	LVY71	*MAT*a/α; adaptive evolution and selection; *isu1-Leu132Phe/isu1-Leu132Phe*	This study

**Table 2:** Fermentation performance of LVY34.4 and LVY41.5 strains.

Strain	Copy number of *xylA*	Condition	Xylose consumption rate (g/g^−1^ h^−1^)	Ethanol production rate (g/g^−1^ h^−1^)	Ethanol yield (g/g^−1^)	Xylitol yield (g/g^−1^)	Glycerol yield (g/g^−1^)
LVY34.4	36	Semi-anaerobic batch, synthetic medium (YPX30)	1.32	0.62	0.46 ± 0.02	0.005 ± 0.00	0.01 ± 0.00
LVY41.5	26	Semi-anaerobic batch, synthetic medium (YPX30)	1.03	0.45	0.45 ± 0.02	0.006 ± 0.00	0.01 ± 0.00
