# Supplementary material for: Unraveling the genetic basis of xylose consumption in engineered Saccharomyces cerevisiae strains
Source: Sci Rep. 2016 Dec 21;6:38676. doi: 10.1038/srep38676 (PMC5175268; doi:10.1038/srep38676)
Supplement: Supplementary Material [file srep38676-s1.pdf]

Supplementary Material Santos *et al.* 2016

Unraveling the genetic basis of xylose consumption in engineered *Saccharomyces cerevisiae* strains

Leandro Vieira dos Santos[1,2], Marcelo Falsarella Carazzolle[1], Sheila Tiemi Nagamatsu[1], Nádia M. V. Sampaio[3], Ludimila Dias Almeida[2], Renan Augusto Siqueira Pirolla[2], Guilherme Borelli[1], Thamy Lívia Ribeiro Corrêa[1], Juan Lucas Argueso[3], Gonçalo Amarante Guimarães Pereira[§1,2]

[1] Laboratório de Genômica e Expressão, Departamento de Genética e Evolução, UNICAMP, Campinas, São Paulo 13083-970, Brazil.

[2] GranBio / BioCelere, Campinas, Brazil.

[3]Department of Environmental and Radiological Health Sciences, Colorado State University, Fort Collins-CO, 80523-1618, USA

[§]To whom correspondence should be addressed. E-mail: goncalo@unicamp.br

Supplementary Figures

Supplementary Figure S1

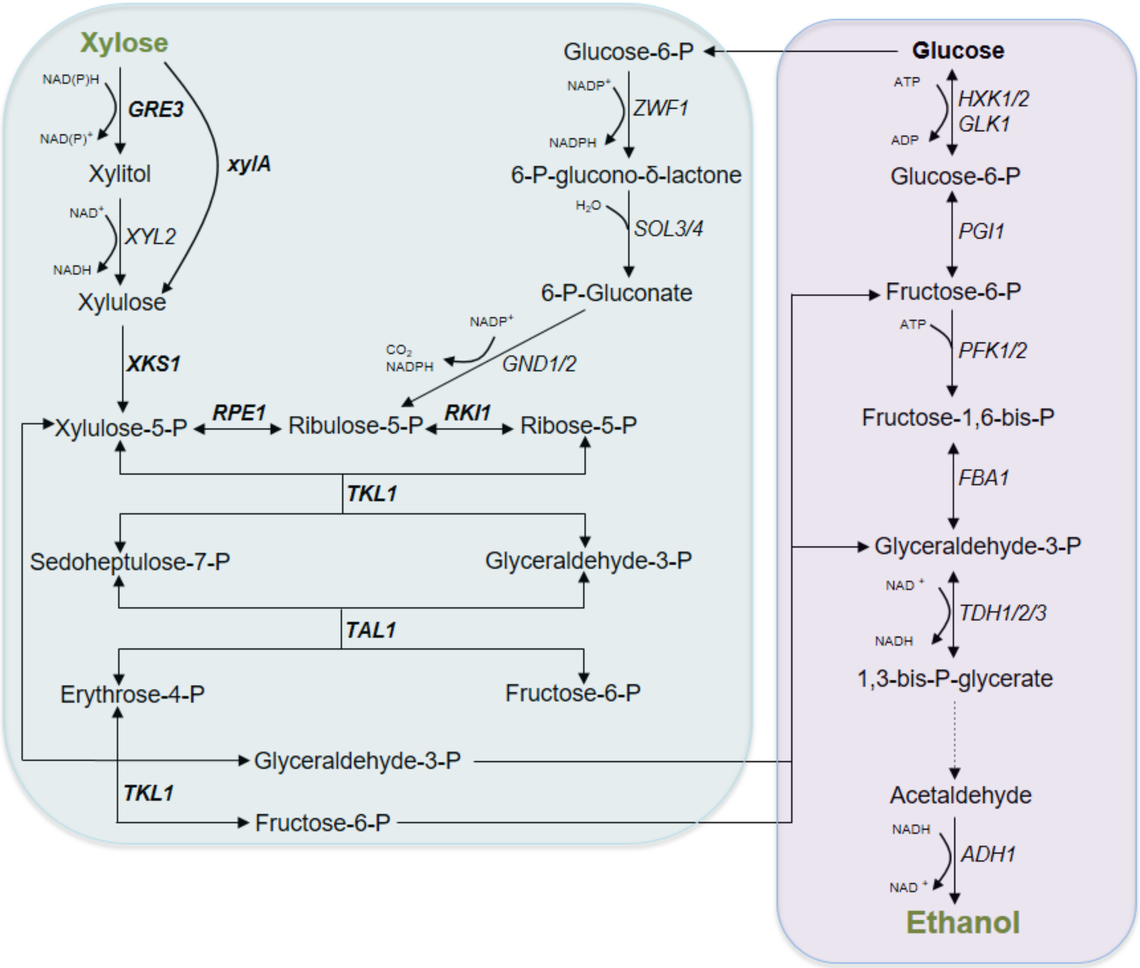

Figure S1. Metabolic network overview of central carbon pathways.
Overview of the xylose and glucose metabolic pathways to ethanol. Xylose pathway, the oxidative and non-oxidative branch of the pentose phosphate pathway are shaded in blue. Glucose fermentation is shaded in purple. All genes used to engineer *S. cerevisiae* in this study are highlighted in bold. The cofactors, ATP and CO_2 are showed. *xylA*, xylose isomerase; *GRE3*, aldose reductase; *XYL2*, xylitol dehydrogenase; *XKS1*, xylulokinase; *RPE1*, ribulose-5-phosphate 3-epimerase; *RPI1*, ribulose-5-phosphate isomerase; *TAL1*, transaldolase; *TKL1*, transketolase; *ZWF1*, glucose-6-phosphate dehydrogenase; *SOL3/4*, 6-phosphogluconolactonase; *GND1/2,* 6-phosphogluconate dehydrogenase; *HXK1/2*, hexokinase; *GLK1*, Glucokinase; *PGI1*, phosphoglucose isomerase; *PFK1/2*, phosphofructokinase; *FBA1*, fructose 1,6-bisphosphate aldolase; *TDH1/2/3*, Glyceraldehyde-3-phosphate dehydrogenase; *ADH1*, alcohol dehydrogenase.

Supplementary Figure S2A

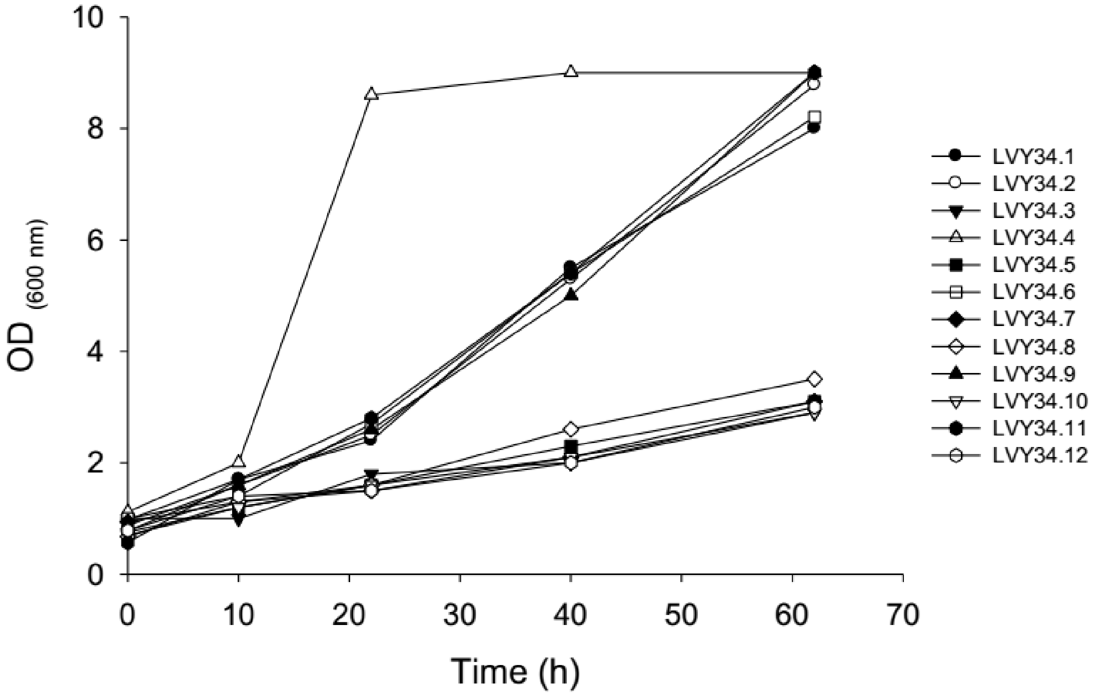

Supplementary Figure S2B

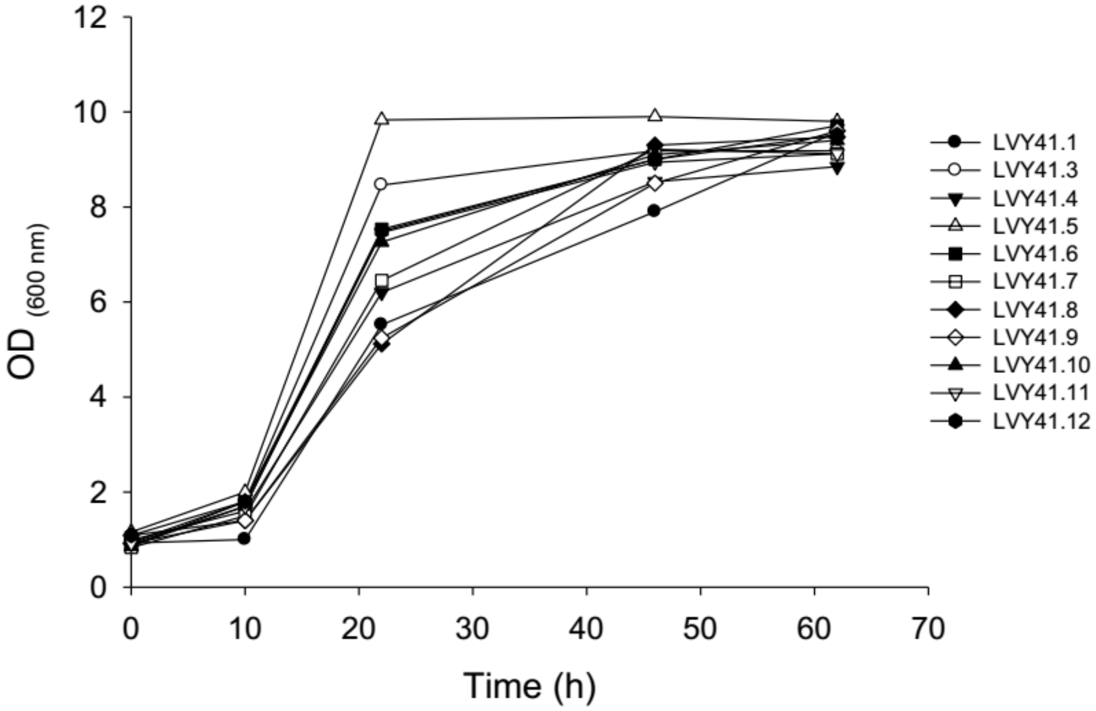

Figure S2. Isolation of the xylose fermenting strains LVY34.4 and LVY 41.5.

Analyses of evolved strains obtained after xylose adaptation. Names of clones are indicated in the legend at right. From the heterogeneous population, LVY34.4 **(A)** and LVY41.5 **(B)** presented the highest growth rates and were selected as the best xylose fermenting strains.

Supplementary Figure S3

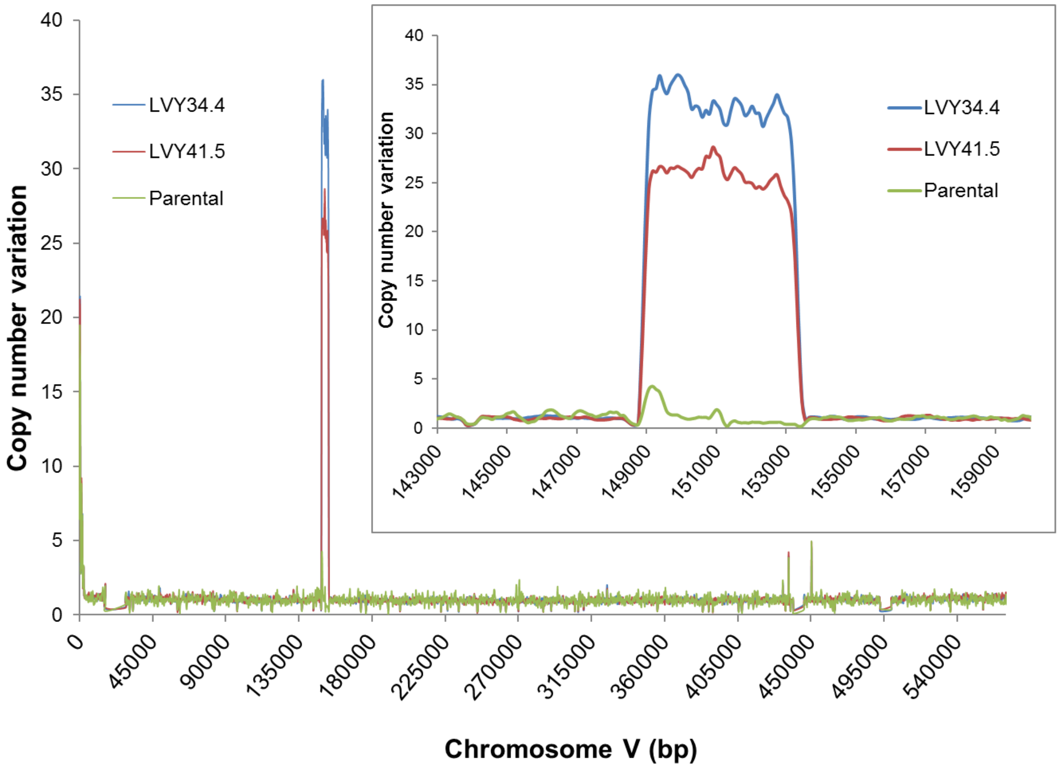

Figure S3. Copy Number Variation (CNV) at chromosome V.
Normalized read-depth coverage representing the copy number variation as a function of Chr05 position for parental LVY27 (green), LVY34.4 (blue) and LVY41.5 (red). The insert contains the region next to *CEN5* where the cassette from plasmid pOXylATy1 harboring the *xylA* gene was integrated. This analysis revealed amplification of the cassette in both evolved strains, with approximately 36 copies in LVY34.4 and 26 copies in LVY41.5.

Supplementary Figure S4

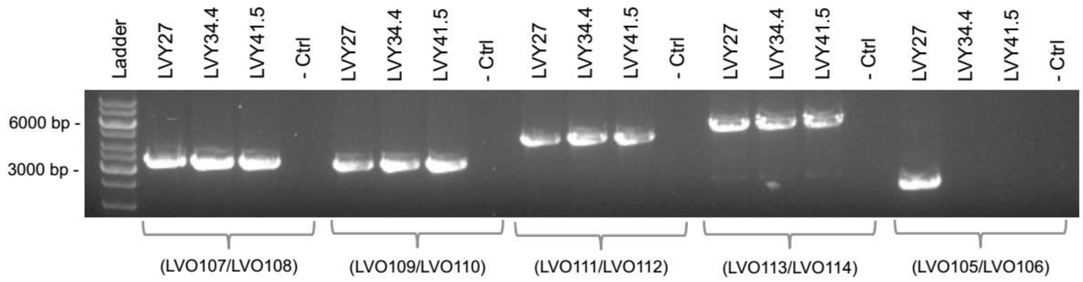

Figure S4. Stability of the transformants after xylose adaptive evolution.
Each PCR reaction was performed with specific primers for genes from the xylose pathway and regions outside the integrated cassettes. LVO107/LVO108 and LVO109/LVO110 were used to confirm *XKS1* cassette, which was integrated next to *CEN2* and *CEN8*, respectively. LVO111/LVO112 and LVO113/LVO114 confirmed the genes from the PPP pathway, inserted next to *CEN12* and *CEN13*, respectively. LVO105/LVO106 pair amplifies the region containing the first *xylA* gene from the plasmid pOXylA next to *CEN5*. The large size of this region in LVY34.4 and LVY41.5 genomes due to the tandem multiplication of this gene precluded the amplification of this region in these strains. Only the parental strain had a positive amplification because it has only one copy of this cassette. 1 Kb DNA ladder GeneRuler; (- Ctrl) control reaction without DNA.

Supplementary Figure S5

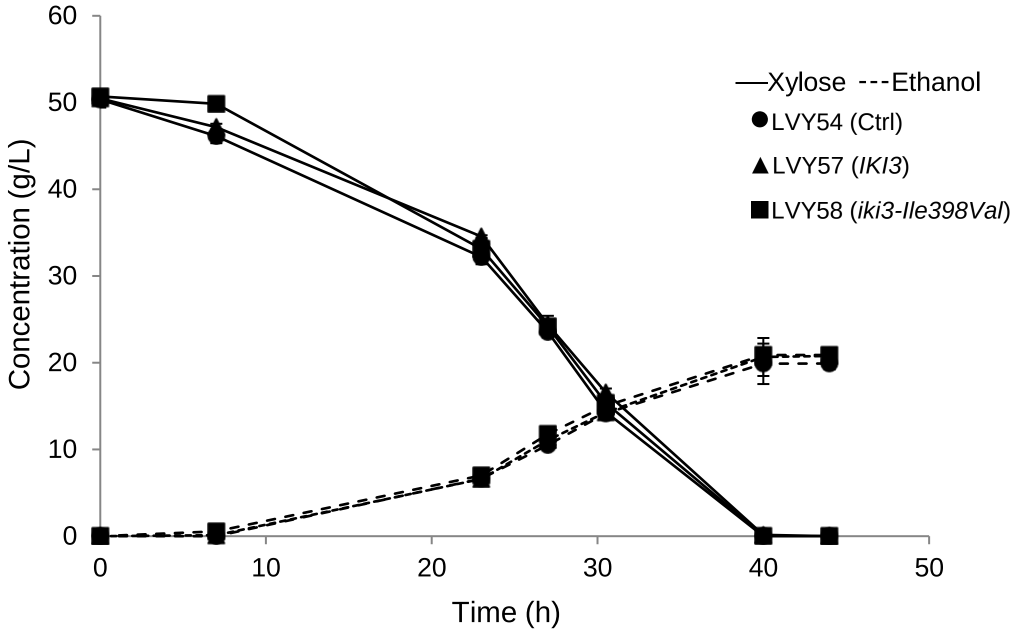

Figure S5. Fermentative performance of the transformants with *IKI3* SNP in xylose medium.

(A) Analysis of the effect of *iki3-Ile398Val* mutation on fermentative performance of control LVY54 (■); LVY57 (●) and LVY58EV (▲). Replacement of *iki3* mutation resulted in no difference in xylose fermentation. Strains were cultivated in YP medium supplemented with xylose in a comparative batch fermentation. The fermentations were performed in triplicate and error bars represent standard deviation from the average of values.

Supplementary Figure S6

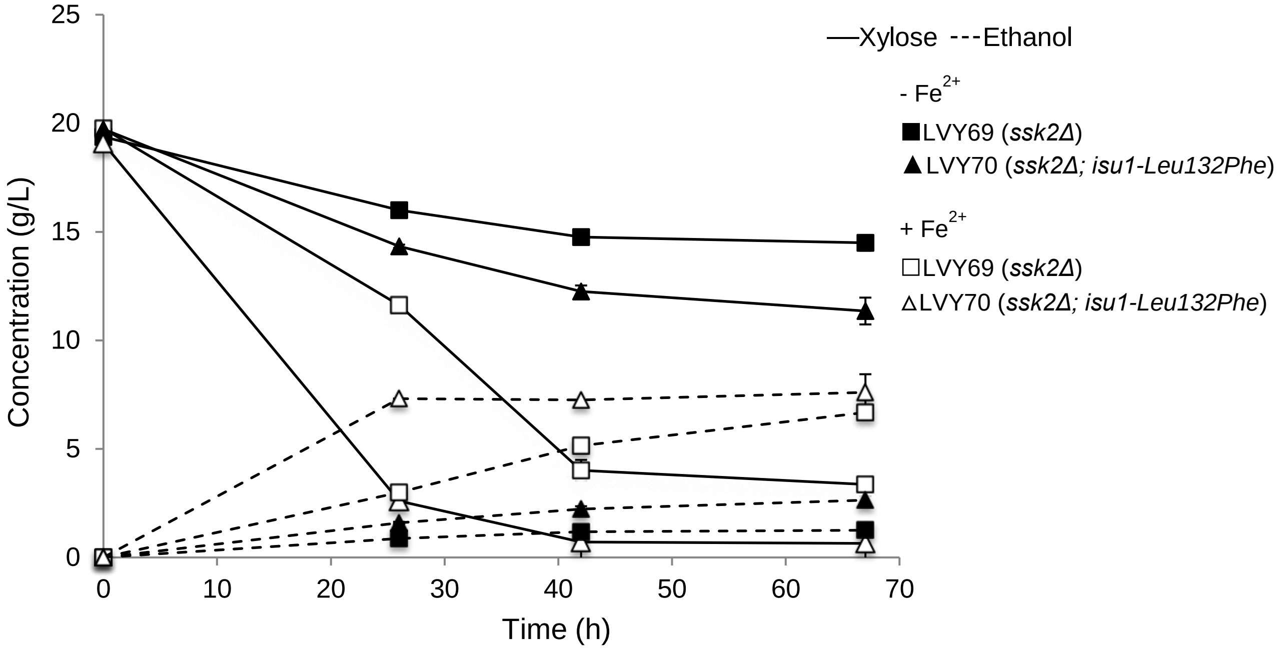

Figure S6. Iron supplementation effect on xylose fermentation.

Effect of iron supplementation in non-evolved strains with *ssk2* deletion. Iron supplementation enabled a faster xylose consumption and ethanol production. Combination with *ISU1* mutation enable a faster consumption by LVY70. Strains were cultivated in YNBX medium supplemented with iron ion. Fermentation was performed in triplicate and error bars represent standard deviation from the average of values.

Supplementary Figure S7

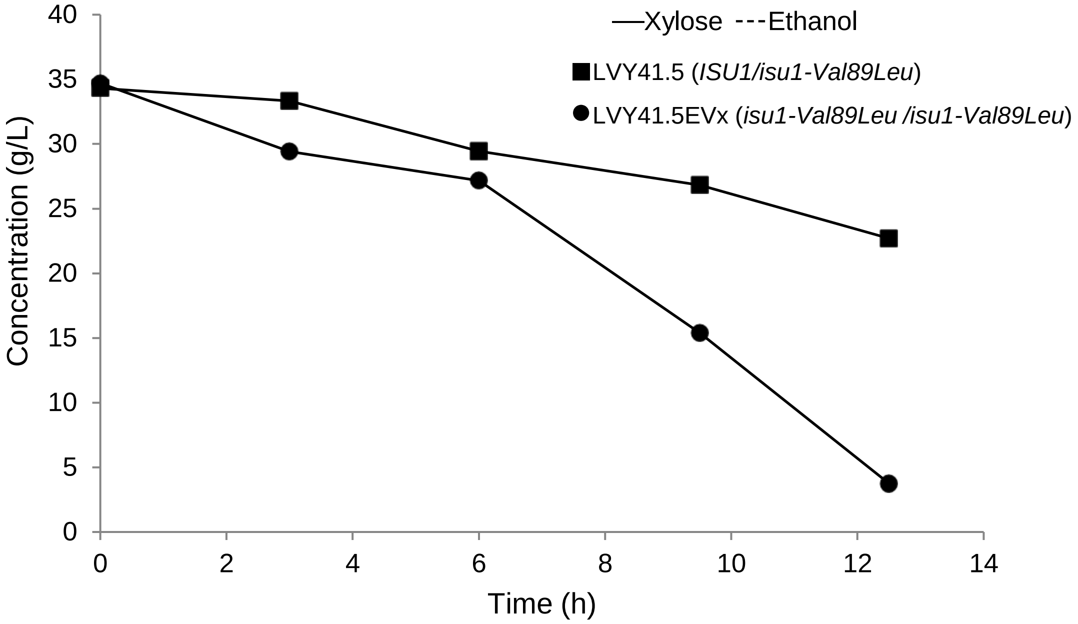

Figure S7. Xylose consumption improvement by second round of evolution in LVY41.5.

Strains were cultivated in YP medium supplemented with xylose. The diploid LVY41.5 (■) was submitted to a second stage of evolution in xylose, resulting in LVY41.5EVx (●). After the second round of evolution, LVY41.5EVx showed higher xylose consumption rate. The fermentation was performed in triplicate and error bars represent standard deviation from the average of values and are not visible when smaller than the symbol size.

Supplementary Tables

Supplementary Table S1. Plasmids used in the study

Plasmid	Parent plasmid	Relevant features	Reference/source
pSH65		Cre recombinase, zeocin resistance	51
pFA6KanMX4		KanMX4 - G418 resistance	52
pAG32		HphMX4 - Hygromycin B resistance	53
pRS304		*TRP1*	55
pRS426		Multi copy plasmid, *URA3*	56
pOXylA	pRS304	pRS426; *pTDH1-XylA-tTDH1, loxP-URA3-loxP*	This study
pOXylA2	pRS426	pRS426; *pTDH1-XylA-tTDH1*	This study
pOXylATy1	pRS304	pRS304; δ *pTDH1-XylA-tTDH1* δ, *LEU2*	This study
pScXKS1	pRS304	pRS304; *pADH1-XKS1-tADH1, loxP-URA3-loxP*	This study
pScTAL1RKI1	pRS304	pRS304; *pTDH1-TAL1-tTDH1, loxP-URA3-loxP*, *pPGK1-RKI1-tPGK1*	This study
pScTKL1RPE1	pRS304	pRS304; *pTDH1-TKL1-tTDH1, loxP-URA3-loxP, pPGK1-RPE1-tPGK1*	This study

Supplementary Table S2. Primers used in the study

Primers ID	Sequence 5'to 3'	Description
JLMo9	AGTCACATCAAGATCGTTTATGG	29; mating type
JLMo10	GCACGGAATATGGGACTACTTCG	29; mating type
JLMo11	ACTCCACTTCAAGTAAGAGTTTG	29; mating type
LVO01	ACCCAACTGCACAGAACAAAAACCTGCAGGAAACGAAGATAAATCTGTGGCTGTGGTTTCAG	Deletion of *URA3*
LVO02	TGTGAGTTTAGTATACATGCATTTACTTATAATACAGTTTTGTGGAGGGTAATTCTGCTTA	Deletion of *URA3*
LVO03	ATAACTTCGTATAGCATACATTATACGAAGTTATGGTCCATAAAGCTTTTCAAT	Plasmids assemble
LVO04	ATAACTTCGTATAATGTATGCTATACGAAGTTATAGAAATCATTACGACCGAGA	Plasmids assemble
LVO19	TTTGTTTTGTGTGTAAATTTAG	pOXylA assemble
LVO20	ATAAAGCAATCTTGATGAGG	pOXylA assemble
LVO21	ATGACTAAAGAATATTTTCCAAC	pOXylA assemble
LVO22	TTATTGGTACATGGCAACA	pOXylA assemble
LVO23	AGCTATTGTTGCCATGTACCAATAAATAAAGCAATCTTGATGAGG	pOXylA assemble
LVO24	TAGTTGGAAAATATTCTTTAGTCATTTTGTTTTGTGTGTAAATTTAG	pOXylA assemble
LVO25	TATAATGTATGCTATACGAAGTTATTTGCAAAGGCAGCAATATAT	pOXylA assemble
LVO26	AAATTTAGAAGACAGCAAGACTAACCATTTGATAGCGTAAATGTATATGCTCATTTACAC	pOXylA assemble
LVO27	CTTTCTCTTCCTATCTGTCCAACATTTCTAGATAAGCCTTACATAACTTCGTATAATGTATGC	pOXylA assemble
LVO28	AGCTCCACCGCGGTGGCGGCCGCTCTAGAACTAGTGGATCCCAGAGTACGACGTACAAAA	pOXylA assemble
LVO29	GATAAGCTTGATATCGAATTCCTGCAGCCCGGGGGATCCCATATGTATATGCATTCATCG	pOXylA assemble
LVO34	ATATAGAAGCAAATAGTTGTCAGTGCAATCCTTCAAGACGATCACTATAGGGCGAATTGG	Deletion of *GRE3*
LVO33	GTAAAAATTTATACACATATACAGCATCGGAATGAGGGAAATCTCAAGCTATGCATCCAA	Deletion of *GRE3*
LVO34	ATGTTGTGTTCAGTAATTCAG	ScXKS1 assemble
LVO35	TTAGATGAGAGTCTTTTCCAG	ScXKS1 assemble
LVO36	GTCTCTGAATTACTGAACACAACATTGTATATGAGATAGTTGATTGTAT	ScXKS1 assemble
LVO37	CGAACTGGAAAAGACTCTCATCTAAGCGAATTTCTTATGATTTATGA	ScXKS1 assemble
LVO38	TATAATGTATGCTATACGAAGTTATTACAATTGGGTGAAATGG	ScXKS1 assemble
LVO39	GAGCAAATGCCTGCAAATCGCTCCCCATTTCACCCAATTGTACACTATAGGGCGAATTGG	ScXKS1 assemble
LVO40	CCTTGTAGCTTGCCAGATCTTCTTGCTTATTCTTAAGCTCTTTTCCGGGTGTACAATATG	ScXKS1 assemble
LVO41	AGTGTTCCCGATGTCAAGCAGTCTTATACAGTACTCCCTATAACTTCGTATAATGTATGC	ScXKS1 assemble
LVO42	CTCCACCGCGGTGGCGGCCGCTCTAGAACTAGTGGATCCTTCAAACTAGGAGTTTGTTGA	ScXKS1 assemble
LVO43	TAAGCTTGATATCGAATTCCTGCAGCCCGGGGGATCCAAGCTTTCTATTAGTCATTCTTC	ScXKS1 assemble
LVO50	TGAGGGTGTATCACCACCTCTCTACTATTATCTAGATGCTTTTTCCGGGTGTACAATATG	ScXKS1 assemble
LVO51	CTATATTACTCGCGCCAACTACCATTCGACGCAAGGAACAAACTCAAGCTATGCATCCAA	ScXKS1 assemble
LVO54	ATGTCTGAACCAGCTCAAA	pScTAL1RKI1 assemble
LVO55	TTAAGCGGTAACTTTCTTTTC	pScTAL1RKI1 assemble
LVO56	GTAGTCCATAGGTACGATCATTTCCTTTTTAACCGTTAAAATGTATATGCTCATTTACAC	pScTAL1RKI1 assemble
LVO57	GTTTCTTTTGAGCTGGTTCAGACATTTTGTTTTGTGTGTAAATTTAG	pScTAL1RKI1 assemble
LVO58	GATTGAAAAGAAAGTTACCGCTTAAATAAAGCAATCTTGATGAGG	pScTAL1RKI1 assemble
LVO59	TATAATGTATGCTATACGAAGTTATTTGCAAAGGCAGCAATATAT	pScTAL1RKI1 and pScTKL1RPE1 assemble
LVO60	TATAGCATACATTATACGAAGTTATTACTGTAATTGCTTTTAGTTGTG	pScTAL1RKI1 and pScTKL1RPE1 assemble
LVO61	ATGGCTGCCGGTGTCC	pScTAL1RKI1 assemble
LVO62	TCACTTTTCCGTAACTTCAACAC	pScTAL1RKI1 assemble
LVO63	CAATTTTTGGGACACCGGCAGCCATTGTTTTATATTTGTTGTAAAAAGTA	pScTAL1RKI1 assemble
LVO64	TAGTGTTGAAGTTACGGAAAAGTGAATTGAATTGAATTGAAATC	pScTAL1RKI1 assemble
LVO65	TAAGAGCATTCGCTGGTAAACAAAATACATCTTGTCGCTTGCAAGGCATTAAAAGAGGAG	pScTAL1RKI1 assemble
LVO66	CTCCACCGCGGTGGCGGCCGCTCTAGAACTAGTGGATCCGAAGCTCTACCTAAAGGTATA	pScTAL1RKI1 assemble
LVO67	TAAGCTTGATATCGAATTCCTGCAGCCCGGGGGATCCCTAGAAATTTCGTTCATCTAG	pScTAL1RKI1 assemble
LVO74	GATACATGTCAAGTAAGCCAGCTTTTGATAAAACTTAAAAATGTATATGCTCATTTACAC	pScTKL1RPE1 assemble
LVO75	GCTAGCTTATCAATGTCAGTAAATTGAGTCATTTTGTTTTGTGTGTAAATTTAG	pScTKL1RPE1 assemble
LVO76	TGACAAGCTAATTTCTCCTTTGAAAAAAGCTTTCTAAATAAAGCAATCTTGATGAGG	pScTKL1RPE1 assemble
LVO77	GCAAGGATACTGGGAGCTATAATTGGTTTGACCATTGTTTTATATTTGTTGTAAAAAGTA	pScTKL1RPE1 assemble
LVO78	TTTTGAAAGCTATTCACTTGGTACATCCGCGGTTTACCAATTAAGGCATTAAAAGAGGAG	pScTKL1RPE1 assemble
LVO79	AGCTCCACCGCGGTGGCGGCCGCTCTAGAACTAGTGGATCCTAGCAAATAACTGCCCATA	pScTKL1RPE1 assemble
LVO80	ATGACTCAATTTACTGACAT	pScTKL1RPE1 assemble
LVO81	TTAGAAAGCTTTTTTCAAAG	pScTKL1RPE1 assemble
LVO82	GCAAGGATACTGGGAGCTATAATTGGTTTGACCATTGTTTTATATTTGTTGTAAAAAGTA	pScTKL1RPE1 assemble
LVO83	ATGGTCAAACCAATTATAG	pScTKL1RPE1 assemble
LVO84	CTAATCTAGCAAATCTCTAGA	pScTKL1RPE1 assemble
LVO85	AGTCTCGAAGGAATTGCGTTCTAGAGATTTGCTAGATTAGATTGAATTGAATTGAAATC	pScTKL1RPE1 assemble
LVO86	ATAAGCTTGATATCGAATTCCTGCAGCCCGGGGGATCCAATTATTTTTACAGGTGCC	pScTKL1RPE1 assemble
LVO93	GCATCACAATACTTGAAGTTGACAATATTATTTAAGGACCTACACTATAGGGCGAATTGG	Deletion of *LEU2*
LVO94	TTGGATGCATAGCTTGAGCCTCCTCTTCAGTACTAAATCTACCAATACCAAATCTGATGG	Deletion of *LEU2*
LVO95	CTGAGAGATTGGTGAATT	pOXylATy1 assemble
LVO96	GAATAAAAATCAACTATCGTC	pOXylATy1 assemble
LVO97	AGCTCCACCGCGGTGGCGGCCGCTCTAGAACTAGTGGATCCCTGAGAGATTGGTGAATT	pOXylATy1 assemble
LVO98	TGTGACTACTAGTTTATAGACGATAGTTGATTTTTATTCAATGTATATGCTCATTTACAC	pOXylATy1 assemble
LVO99	AATATTAGGTATGTGGATATACTAGAAGTTCTCCTCGATTGCAAAGGCAGCAATA	pOXylATy1 assemble
LVO100	TCGAGGAGAACTTCTAGTA	pOXylATy1 assemble
LVO101	TCGACTACGTCGTAAGG	pOXylATy1 assemble
LVO102	GAATGCCTACAATCATCTCAAAATTCACCAATCTCTCAGTCGACTACGTCGTAAGG	pOXylATy1 assemble
LVO103	ATAAGCTTGATATCGAATTCCTGCAGCCCGGGGGATCCGAATAAAAATCAACTATCGTC	pOXylATy1 assemble
LVO105	TCAATGTCTGTATTCATCGAT	Check integration *xylA* near *CEN5*
LVO106	GATGCCGCTATAATGGAA	Check integration *xylA* near *CEN5*
LVO107	TGAGACGATTTAGAGTAAGGT	Check integration *XKS1* near *CEN2*
LVO108	AAGCTTTCTATTAGTCATTCTTC	Check integration *XKS1* near *CEN2*
LVO109	CTCAAGCCCAAATCTCTT	Check integration *XKS1* near *CEN8*
LVO110	TACTGAAAGCAATTTTGCG	Check integration *XKS1* near *CEN8*
LVO111	ATGTGCTCTCCTCTCCATTC	Check integration *TAL1* and *RKI1* near *CEN12*
LVO112	CGTTGATATTAGCATACACCAG	Check integration *TAL1* and *RKI1* near *CEN12*
LVO113	TGAGAAGTTCTGGCTACCT	Check integration *TKL1* and *RPE1* near *CEN13*
LVO114	TTGTATGTATGGGATGATAAC	Check integration *TKL1* and *RPE1* near *CEN13*
LVO211	AAGCGATGCCTAGGCAAGAAACAGTACAAATGCCTAATGGCTTGCGGGAATTCGATATAAC	Deletion of *IKI3*
LVO212	AGCACTATAGACAGTAATTTATATAACTAAGAAAATGGTATGCCGCGAATTCACTAGTGAT	Deletion of *IKI3*
LVO213	GTTCCAGTTCCTGTTGTAGC	*IKI3* restoration
LVO214	TGTTTCTTCATGTAATTATTCAA	*IKI3* restoration
LVO218	AATTGAATAAGGAAAACACAACACATAACACATATTTAACCTGGCGGGAATTCGATATAAC	Deletion of *ISU1*
LVO219	TGAGGGTTTGATCTTGTTCTTGTCCCGGTTATCTTCTTATTCACGCGAATTCACTAGTGAT	Deletion of *ISU1*
LVO220	GAGAAGTGGTTCCTTAACCTTAAT	*ISU1* restoration and sequencing
LVO221	TGATGCCATATACCACACATG	*ISU1* restoration and sequencing
LVO222	GGAGCATGGTCGTATTGAAGATGAAATGGTCACTCAAGTCTAGCCAGCTGAAGCTTCGTA	Deletion of *SSK2*
LVO223	GTGATCATTTCTAATACCACGCAGCCTAACGACCAAACATCGTAGGGAGACCGGCAGATC	Deletion of *SSK2*
JAO1480	CCTGTCTTGTTGGGTCCAGT	qPCR chr05, 09 and 16
JAO1481	TTGAACTTCAGTGGCACCAG	qPCR chr05, 09 and 16
JAO1482	TGGGACAATGGGTTAGTCGT	qPCR chr05, 09 and 16
JAO1483	ATTCTGGCCAACTCTGCTGT	qPCR chr05, 09 and 16
JAO1484	TCAAACCTTTGGGTTCCAAG	qPCR chr05, 09 and 16
JAO1485	CCCGATGGACAAAGTGTCTT	qPCR chr05, 09 and 16

Supplementary Table S3. Overall data for genome sequencing and assembly

Strain	Number of paired-end reads	Genome coverage	Assembled contigs	N50 (Kbp)	Total of assembly (Kbp)
**LVY27**	1,087,889	56,499	226	159.4	11,553.1
**LVY34.4**	2,711,916	139,575	213	160.4	11,657.9
**LVY41.5**	2,482,732	128,220	232	172.9	11,617.8

Supplementary Table S4. SNPs and Indels identified in the evolved strains by genomic analysis

Gene ID	Name	REF	ALT	Consequence	Amino acid change	Chr/position	LVY34.4 allele frequency	LVY41.5 allele frequency
YCL021W-A	YCL021W-A	T	C	downstream gene variant	-	III/90807	1	0.5
		G	T	downstream gene variant	-	III/90857	1	-
YCR037C	*PHO87*	G	A	synonymous variant	-	III/196889	-	0.5
YCR084C	*TUP1*	G	A	synonymous variant	-	III/261884	-	0.5
YLR176C	*RFX1*	G	T	upstream gene_variant	-	XII/510494	-	0.5
YLR384C	*IKI3*	T	C	missense variant	Ile398Val	XII/891709	1	1
YNR031C	*SSK2*	G	T	stop gained	Glu1451Ter	XIV/681083	-	0.5
YNR038W/ YNR039C	*DBP6/ZRG17*	TA	T	downstream gene variant	-	XIII/697545	-	0.5
YPL135W	*ISU1*	G	C	missense variant	Val89Leu	XVI/297817	-	0.5
		G	T	missense variant	Leu132Phe	XVI/297948	1	-

Supplementary material

Codon optimized sequence of *xylA* gene from *Orpinomyces* sp.

ATGACTAAAGAATATTTTCCAACTATTGGTAAAATTAGATTTGAAGGTAAAGATTCTAAGAA
TCCAATGGCCTTCCATTACTATGATGCTGAAAAAGAAGTCATGGGTAAGAAAATGAAAGATT
GGTTAAGATTTGCCATGGCCTGGTGGCATACTTTGTGCGCCGATGGTGCTGACCAATTCGGT
GTTGGTACTAAGTCTTTTCCATGGAATGAAGGTACTGACCCAATTGCTATTGCCAAACAAAA
GGTTGATGCTGGTTTTGAAATTATGACCAAATTGGGTATTGAACATTATTGTTTCCACGATG
TTGATTTAGTTTCTGAAGGTAATTCTATTGAAGAATATGAATCTAACTTGAAACAAGTTGTT
GCTTACTTGAAACAAAAGCAACAAGAAACTGGTATTAAATTATTGTGGTCTACTGCCAATGT
TTTTGGTAATCCAAGATATATGAACGGTGCCTCTACTAATCCAGACTTTGATGTCGTCGCCA
GAGCTATTGTTCAAATTAAGAACGCCATGGACGCCGGTATTGAATTGGGTGCTGAAAACTAC
GTCTTCTGGGGTGGTAGAGAAGGTTATATGTCATTGTTAAACACTGACCAAAAAAGAGAAAA
GGAACATATGGCTACTATGTTGACTATGGCTAGAGATTACGCTAGATCTAAAGGTTTTAAGG
GTACTTTCTTAATTGAACCAAAACCAATGGAACCAACCAAGCATCAATATGACGTTGATACT
GAAACTGTTATTGGTTTCTTGAGAGCTCACAATTTAGACAAAGACTTTAAGGTCAACATTGA
AGTTAATCACGCTACTTTAGCTGGTCATACTTTCGAACACGAATTGGCCTGTGCTGTTGATG
CTGGTATGTTAGGTTCTATTGATGCTAACAGAGGTGACTATCAAAATGGTTGGGACACTGAT
CAATTCCCAATTGATCAATATGAATTGGTCCAAGCTTGGATGGAAATTATCAGAGGTGGTGG
TTTTGTTACTGGTGGTACCAACTTCGATGCCAAAACTAGAAGGAACTCTACCGATTTAGAAG
ATATTATCATTGCTCATATTTCTGGTATGGATGCCATGGCTAGAGCTTTGGAAAATGCTGCC
AAGTTATTGCAAGAATCTCCATATTGTAATATGAAAAAGGAAAGATACGCTTCTTTTGACTC
TGGTATTGGTAAAGACTTTGAAGATGGTAAGTTAACTTTGGAACAAGTTTACGAATATGGTA
AAAAGAATGGTGAACCAAAAGTTACTTCTGGTAAGCAAGAATTATATGAAGCTATTGTTGCC
ATGTACCAATAA
